# Hypoxia‐inducible factor 2α drives hepatosteatosis through the fatty acid translocase CD36

**DOI:** 10.1111/liv.14519

**Published:** 2020-06-10

**Authors:** Esther Rey, Florinda Meléndez‐Rodríguez, Patricia Marañón, Miriam Gil‐Valle, Almudena G. Carrasco, Mar Torres‐Capelli, Stephania Chávez, Elvira del Pozo‐Maroto, Javier Rodríguez de Cía, Julián Aragonés, Carmelo García‐Monzón, Águeda González‐Rodríguez

**Affiliations:** ^1^ Unidad de Investigación Hospital Universitario Santa Cristina Instituto de Investigación Sanitaria del Hospital Universitario de La Princesa Madrid Spain; ^2^ Unidad de Investigación Hospital Universitario Santa Cristina Instituto de Investigación Sanitaria del Hospital Universitario de La Princesa Universidad Autónoma de Madrid Madrid Spain; ^3^ Centro de Investigación Biomédica en Red de Enfermedades Cardiovasculares (CIBERCV) Madrid Spain; ^4^ Centro de Investigación Biomédica en Red de Enfermedades Hepáticas y Digestivas (CIBEREHD) Madrid Spain; ^5^ Present address: Dpto. Ciencias Básicas de la Salud Universidad Rey Juan Carlos Alcorcón Spain

**Keywords:** CD36, HIF2α, hypoxia, NAFLD, steatosis

## Abstract

**Background & Aims:**

Molecular mechanisms by which hypoxia might contribute to hepatosteatosis, the earliest stage in non‐alcoholic fatty liver disease (NAFLD) pathogenesis, remain still to be elucidated. We aimed to assess the impact of hypoxia‐inducible factor 2α (HIF2α) on the fatty acid translocase CD36 expression and function in vivo and in vitro.

**Methods:**

CD36 expression and intracellular lipid content were determined in hypoxic hepatocytes, and in hypoxic CD36‐ or HIF2α ‐silenced human liver cells. Histological analysis, and HIF2α and CD36 expression were evaluated in livers from animals in which von Hippel‐Lindau (*Vhl*) gene is inactivated (Vhl^f/f^‐deficient mice), or both *Vhl* and *Hif2a* are simultaneously inactivated (Vhl^f/f^Hif2α^/f^‐deficient mice), and from 33 biopsy‐proven NAFLD patients and 18 subjects with histologically normal liver.

**Results:**

In hypoxic hepatocytes, CD36 expression and intracellular lipid content were augmented. Noteworthy, *CD36* knockdown significantly reduced lipid accumulation, and *HIF2A* gene silencing markedly reverted both hypoxia‐induced events in hypoxic liver cells. Moreover livers from Vhl^f/f^‐deficient mice showed histologic characteristics of non‐alcoholic steatohepatitis (NASH) and increased CD36 mRNA and protein amounts, whereas both significantly decreased and NASH features markedly ameliorated in Vhl^f/f^Hif2α^f/f^‐deficient mice. In addition, both HIF2α and CD36 were significantly overexpressed within the liver of NAFLD patients and, interestingly, a significant positive correlation between hepatic transcript levels of *CD36* and erythropoietin (*EPO*), a HIF2α ‐dependent gene target, was observed in NAFLD patients.

**Conclusions:**

This study provides evidence that HIF2α drives lipid accumulation in human hepatocytes by upregulating CD36 expression and function, and could contribute to hepatosteatosis setup.

**KEY POINTS**

CD36 knockdown attenuates hypoxia‐induced lipid accumulation in hepatocytes. HIF2α silencing reverts both lipid and CD36 content accumulation in hypoxic hepatocytes and in livers from a murine experimental model which displays NAFLD features due to an overexpression of HIF1 and HIF2. Expression of HIF2α and CD36 is increased in the liver of NAFLD patients.

## 1 INTRODUCTION

Overnutrition is a major contributor to the development of non‐alcoholic fatty liver disease (NAFLD) because a high consumption of saturated fatty acids, cholesterol and fructose along with a low intake of polyunsaturated fatty acids, featuring NAFLD patients, alters hepatic lipid metabolism homeostasis leading to an excessive fat accumulation within the liver which activates inflammation, hepatocellular damage and fibrogenesis.^1^, ^2^ There is extensive clinical and experimental evidence indicating that chronic intermittent hypoxia, featuring a respiratory disorder of growing prevalence worldwide termed obstructive sleep apnoea, could contribute to the progression of NAFLD from simple steatosis, also termed non‐alcoholic fatty liver (NAFL) or hepatosteatosis, to non‐alcoholic steatohepatitis (NASH),^3^, ^4^, ^5^, ^6^ the most clinically relevant form of NAFLD with a significant risk to progress into cirrhosis and hepatocellular carcinoma,^7^, ^8^ as well as increasing the cardiovascular morbidity and mortality and the incidence of extrahepatic cancers.^9^, ^10^

Hypoxia‐inducible factors (HIFs), particularly HIF1α and HIF2α, are master regulators of hypoxia‐induced cellular adaptive responses elicited to restore cell metabolism and survival.^11^ HIFs are implicated in numerous physiological and pathological conditions, and it has been reported that HIF2α promotes NASH in mice,^12^, ^13^ and dysregulates lipid metabolism in HepG2 cells.^14^ Since lipotoxicity due to free fatty acids (FFAs) overload within hepatocytes plays a central role in NAFLD pathophysiology,^15^ and that this process is largely regulated by membrane‐bound FFA transporters, it is conceivable that HIFs might contribute to NAFLD pathogenesis by upregulating the expression and function of FFA transporters in the membrane of hepatocytes.

Among membrane‐bound FFA transporters, the fatty acid translocase CD36 (CD36) is the best characterized.^16^ CD36 functions as a high affinity receptor for long‐chain FFAs contributing under excessive fat supply to lipid accumulation and metabolic dysfunction.^17^ This FFA receptor is involved in several aspects of lipid metabolism including fat taste perception, fat intestinal absorption and FFA utilization by muscle, adipose tissues and liver.^18^ Regarding the latter, hepatic CD36 expression is normally weak but it increases by a number of different stimuli such as cytokines or insulin.^18^, ^19^ Noteworthy, experimental studies have demonstrated that CD36 plays a key role in the hepatosteatosis setup in rodents ^20^, ^21^ and, more interestingly, NAFLD patients present high hepatic CD36 mRNA levels,^22^ and this FFA transporter is largely overexpressed in the plasma membrane of hepatocytes.^23^ While there are evidences that HIF1α upregulates CD36 expression and function in retinal epithelial cells and macrophages,^24^, ^25^ whether HIF2α is able to regulate CD36 gene expression in hepatocytes still remains to be elucidated.

Therefore, the aim of the present study was to determine the impact of HIF2α on CD36 expression and function as well as on lipid content in hepatocytes submitted to hypoxic conditions, in livers from genetically‐modified mice in which von Hippel‐Lindau (*Vhl*) gene is inactivated (Vhl^f/f^‐deficient mice), a murine experimental model which displays NAFLD features due to an overexpression of HIF1 and HIF2, in livers from mice in which both *Vhl* and *Hif2a* are simultaneously inactivated (Vhl^f/f^Hif2α^f/f^‐deficient mice), and in livers from patients with biopsy‐proven NAFLD.

## 2 MATERIALS AND METHODS

### 2.1 Cell culture and treatment

Human hepatoma Huh7 cells and AML12 (alpha mouse liver 12) hepatocyte cell line were purchased from the American Type Culture Collection (ATCC, Manassas, VA). Huh7 cells were cultured in Dulbecco's modified Eagle's medium (DMEM) containing high glucose, penicillin/streptomycin and 10% fetal bovine serum, and AML12 hepatocytes were cultured in DMEM:F12 medium containing high glucose, penicillin/streptomycin and 10% fetal bovine serum, supplemented with 10 µg/mL insulin, 5.5 µg/mL transferrin, 5 ng/mL selenium and 40 ng/mL dexamethasone. The cells were submitted to normoxic (21% O_2_) or hypoxic conditions (1% O_2_) in a hypoxic chamber InvivO2 200 (Ruskinn Technology Ltd.) for 36 hours.

### 2.2 **Short hairpin CD36 or HIF2**α** knockdown**

Human scrambled (shControl, shC), CD36 shRNA or HIF2α shRNA lentiviral particles (Dharmacon, Madrid, Spain) were used to produce stable CD36 or HIF2α knockdown in Huh7 cells. Proliferating cells were co‐incubated with lentiviral transducing particles in culture media containing polybrene (Santa Cruz Biotechnology Inc, Heidelberg, Germany) for 24 hours, and then cultured with 2‐5 µg/mL of puromycin (Santa Cruz Biotechnology, Inc). Resistant cells were expanded and examined for *CD36* or *HIF2A* mRNA levels respectively.

### 2.3 Nile Red staining

The cells were fixed with paraformaldehyde 4% for 30 minutes at 4ºC, and resuspended in Nile Red working solution to 0.4 µL/mL (Sigma‐Aldrich Inc). The fluorescence was determined using a flow cytometer Cytomics FC500 MPL^TM^ (Beckman‐Coulter Inc).

### 2.4 Animals

All animal experimentation was conducted in accordance with Spanish and European legislation and approved by the research ethics committee at the Universidad Autónoma de Madrid (UAM) (CEI55‐1002‐A049). Mice used in this study were maintained in light/dark (12h light/12 hours dark), temperature (22°C) and humidity‐controlled rooms, fed with standard chow diet (LASQCdiet®Rod14, LASvendi, Germany) ad libitum, and had free access to drinking water at the animal facilities of the UAM. Vhl^floxed^‐Ubc‐Cre‐ER^T2^ (Vhl^f/f^‐deficient mice), Vhl^floxed^HIF2α^floxed^‐Ubc‐Cre‐ER^T2^ (Vhl^f/f^Hif2α^f/f^‐deficient mice) mice and their corresponding controls (Vhl^floxed^ lacking UBC‐Cre‐ERT2 or Vhl^floxed^HIF2α^floxed^ lacking UBC‐Cre‐ERT2) were generated as described.^26^ Male mice at 4‐5 months of age were used in this study. For gene inactivation, mice were fed ad libitum with Teckland CRD TAM400/CreER tamoxifen pellets (Harland‐Teklad, Valencia, Spain) for 10‐15 days, including control mice, and were later returned to standard chow diet for 15 days. After that, mice were sacrificed and livers were harvested.

### 2.5 Patients

This study included 33 patients with a clinical diagnosis of NAFLD (18 NAFL and 15 NASH), and 18 subjects with normal liver (NL) who underwent a liver biopsy by a percutaneous route during programmed cholecystectomy. Characteristics of the study population are detailed in Table 1. All NAFLD patients and NL subjects studied drank less than 20 g/day of alcohol, were not having potentially hepatotoxic drugs, had no analytical evidence of iron overload, and were seronegative for autoantibodies and for hepatitis B virus, hepatitis C virus and human immunodeficiency virus. This study was performed in agreement with the Declaration of Helsinki, and with local and national laws. The Human Ethics Committee of the Hospital Universitario Santa Cristina approved the study procedures (PI‐688A), and all participants signed an informed written consent before inclusion in the study.

**Table 1:** Characteristics of the study population

Feature	NL (n = 18)	NAFL (n = 18)	NASH (n = 15)
Age (years)	48.2 ± 12.9	54.3 ± 14.9	47.4 ± 11.7
Body mass index (kg/m^2^)	27.1 ± 4.2	29.9 ± 4*	29.4 ± 2.7
Glucose (mg/dL)	91.4 ± 11.1	96.1 ± 9.6	98.6 ± 15.6
Insulin (µU/L)	7.6 ± 6.6	9.6 ± 4.1*	11.5 ± 5.3*
HOMA‐IR	1.7 ± 0.9	2.3 ± 1.1*	2.8 ± 1.4*
Triglycerides (mg/dL)	110 ± 38.8	135.3 ± 39.8*	138.9 ± 40.4*
HDL‐cholesterol (mg/dL)	48.3 ± 10.8	49.5 ± 8.9	46.3 ± 8.9
ALT (IU/L)	17.2 ± 5.9	24.6 ± 9.3**	42.8 ± 18.6***
AST (IU/L)	17.3 ± 3.8	20.3 ± 5.1*	27 ± 11.9***
GGT (IU/L)	26.2 ± 16.6	39.4 ± 29.6*	52.6 ± 35.2***
Steatosis (%)			
Grade 0	100%		
Grade 1		66.6%	
Grade 2		33.3%	50%
Grade 3			50%
Lobular inflammation (%)			
Grade 0	100%	100%	
Grade 1			80%
Grade 2			10%
Grade 3			10%
Ballooning (%)			
Grade 0	100%	100%	
Grade 1			70%
Grade 2			30%
Grade 3			
Fibrosis (%)			
Stage 0	100%	100%	30%
Stage 1			70%
Stage 2			
Stage 3			

Note

Data are presented as mean ± standard deviation or as number of cases (%). Study population: Normal liver (NL) individuals (n = 18), NAFL patients (n = 18) and NASH patients (n = 15). HOMA‐IR, homeostatic model assessment‐insulin resistance; HDL, high‐density lipoprotein; ALT, alanine aminotransferase; AST, aspartate aminotransferase; GGT, gamma‐glutamyltransferase. *P < .05, **P < .01 and ***P < .005, NAFL or NASH vs NL.

### 2.6 Gene expression analysis by real‐time quantitative PCR

Total RNA from cells or liver samples was extracted using TRIzol reagent (Vitro, Sevilla, Spain) and was reverse transcribed using a reverse transcription system (Promega Inc) in a T100TM Thermal Cycler (BioRad Inc, Madrid, Spain) following manufacturer's indications. Quantitative real‐time polymerase chain reaction (qPCR) was performed with StepOnePlusTM Real Time PCR System sequence detector (Thermo Fisher Scientific, Inc, Madrid, Spain) using the SYBR Green method and d(N)6 random hexamer with primers purchased from Metabion (Steinkirchen, Germany). Each sample was run in duplicated and normalized to *36B4* (mouse and human samples) or *HPRT* (cell samples) gene expression. Fold changes were determined using the ΔΔCt method. Primer sequences are detailed in Table 2.

**Table 2:** Primer sequences for RT‐qPCR

Gene	Forward (5’→3’)	Reverse (5’→3’)
m‐*Cd36*	AGATGACGTGGCAAAGAACAG	CCTTGGCTAGATAACGAACTCTG
m‐*Epo*	CAAAGTCAACTTCTATGCTTGGAAAA	CAGGCCTTGCCAAACTTCTATG
m‐*Hif2a*	GAGGAAGGAGAAATCCCGTGA	TATGTGTCCGAAGGAAGCTGA
m‐*Pgk1*	CAGTTGCTGCTGAACTCAAATCTC	CCCACACAATCCTTCAAGAACA
m‐*Vhl*	ATCCCTGAAGAGCCAAAGATGA	TCAGCCCTACCCGATCTTACC
m‐*36b4*	AGATGCAGCAGATCCGCAT	GTTCTTGCCATCAGCACC
h‐*CD36*	ATGTGTGTGGAGAGCGTCAACC	TGAGCAGAGTCTTCAGAGACAGCC
h‐*EPO*	TTCGCAGCCTCACCACTCT	GAGATGGCTTCCTTCTGGGC
h‐*HIF2A*	CTCATCCCTGCGACCATGA	TTCCCAAAACCAGAGCCATT
h‐*PGK1*	TGGCTTCTGGCATACCTGCT	GCTGCTTTCAGGACCACAGCT
h‐*PHD3*	TGCATCACCTGCATCTACTATCTG	CGCAGGATCCCACCATGTA
h‐*VHL*	CGCCGCATCCACAGCTA	TGTGTCCCTGCATCTCTGAAGA
h‐*36B4*	CAGGCGTCCTCGTGGAAGTGAC	CCAGGTCGCCCTGTCTTCCCT
h‐*HPRT*	ATTGTAATGACCAGTCAACAGGG	GCATTGTTTTGCCAGTGTCAA

### 2.7 Preparation of total protein extracts

Liver biopsy samples were homogenized in 16 volumes (w/v) of cold lysis buffer (50 mM Tris‐HCl, 1% Triton X‐100, 2 mM EGTA, 10 mM EDTA acid, 100 mM NaF, 1 mM Na_4_P_2_O_7_, 2 mM Na3VO4, 100 µg/ml phenylmethylsulphonyl fluoride and protease inhibitors). To obtain total cell lysates, at the end of the experiment, attached cells were scraped off and incubated for 10 minutes on ice with RIPA buffer (50 mM Tris HCl, pH 7.4, 1% Triton X‐100, 0.2% sodium dodecyl sulfate (SDS), 1 mM EDTA, 1 mM PMSF and 5 µg/ml leupeptin). Protein extracts were stored at −80ºC after centrifugation.

### 2.8 Extraction of nuclear protein liver extracts

Liver biopsy samples were homogenized in 4 volumes (w/v) of cold buffer A (0.3 M Sucrose solution with protease inhibitors). Samples were centrifuged and the supernatant containing the cytosolic fraction was stored at −80ºC. The pellet containing the nuclear fraction was washed for a total of three times with buffer A. Samples were incubated in rotation for 1 hour with cold buffer B (1% Trion x100, 1% Sodium Deoxycholate, 0.1% SDS, 5 mM EDTA, 200 nM NaCl, 20 mM Tris HCl pH 8 with protease inhibitors). After sonication, cellular debris was removed by centrifugation and the supernatant fraction containing the nuclear fraction was stored at −80ºC.

### 2.9 Western blot analysis

After protein content determination with Bradford reagent, 50 µg of total protein or100 µg of nuclear protein was boiled in Laemmli sample buffer and submitted to 8% SDS‐PAGE gels. Proteins were transferred to Inmunoblot nitrocellulose membrane (BioRad Inc) and, after blocking with 5% non‐fat dry milk, incubated overnight with different antibodies as indicated: anti‐HIF1α (1:1000, 10006421, Cayman Chemical, Hamburg, Germany), anti‐HIF2α (1:1000, ab199, Abcam, Cambridge, UK) and anti‐CD36 (1:1000, NB400‐144, Novus, Abingdon, UK). Immunoreactive bands were visualized using the enhanced chemiluminescence (ECL) Western blotting protocol (BioRad Inc). The anti‐βactin (1:5000, A‐5441, Sigma‐Aldrich Inc) and the anti‐lamin B (1:1000, ab65986, Abcam) antibodies were used as loading control for total and nuclear protein respectively. Densitometric analysis of the bands was performed using Image J software (NIH, Bethesda, MD).

### 2.10 Histopathology assessment

Paraffin‐embedded liver biopsy sections (4 µm thick) were stained with haematoxylin/eosin and evaluated by a single‐blinded hepatopathologist. Steatosis was determined grading percentage involvement by steatotic hepatocytes as follows: grade 0, 0%‐5%; grade 1, >5%‐33%; grade 2, >33%‐66%; and grade 3, >66%, as described by Kleiner et al.^27^ In addition, this scoring system was used to evaluate the degree of lobular inflammation and hepatocellular ballooning. NAFLD activity score was calculated for each liver biopsy studied as described elsewhere.^27^ Histologic diagnosis of liver biopsies from NAFLD patients was classified into two groups: simple steatosis without hepatocellular ballooning nor lobular inflammation, also termed NAFL, and NASH. Minimal criteria for NASH included the combined presence of grade 1 steatosis, lobular inflammation and hepatocellular ballooning with or without fibrosis. As we want to study early stages of NAFLD, only NASH patients with either mild fibrosis (F1) or without fibrosis (F0) were studied and included in the same group. Representative images were taken using an optical microscope Nikon Eclipse E400 (Nikon, Tokyo, Japan).

### 2.11 Oil Red O staining

Cryoprotected liver biopsy sections (5 µm) were stained with an Oil red O (ORO, Sigma‐Aldrich Inc) working solution (60% ORO/isopropanol w:v) and counter‐stained with haematoxylin. Red staining was quantified from the images taken using an optical microscope Nikon Eclipse E400 (Nikon). Image analysis procedures were performed with the FIJI software (NIH, Bethesda, MD). Values were obtained in six different lobular areas where hepatocytes are the predominant cell type. The average value was considered as ORO staining index for each liver biopsy sample, and expressed as arbitrary units which reflect the intensity of the staining.

### 2.12 **HIF2**α** and CD36 immunohistochemistry**

Paraffin‐embedded liver biopsy sections (4 µm thick) were immunostained with a primary rabbit antibody against HIF2α (ab199, Abcam) ^28^ or CD36 (NB400‐144, Novus) diluted to 1:50 and 1:200 respectively, using the DAKO EnVision™+ System (DAKO, Glostrup, Denmark) as described by the manufacturer. Liver tissue area occupied by nuclear HIF2α or CD36‐positive cells was quantified from the images taken using an optical microscope Nikon Eclipse E400 (Nikon). Image analysis procedures were performed with the FIJI software (NIH). Values were obtained in six different lobular areas where hepatocytes are the predominant cell type. The average value was considered as nuclear HIF2α or CD36 expression index for each liver biopsy sample, and expressed as percentage of positive nuclei or arbitrary units respectively, which reflects the intensity of the immunostaining.

### 2.13 CD36 and N‐cadherin immunofluorescence

Paraffin‐embedded liver biopsy sections (4 μm thick) were co‐incubated with an anti‐CD36 antibody (NB400‐144, Novus) and anti‐N‐cadherin antibody (BP1‐48309, Novus), diluted to 1:200 and 1:25 respectively, following with the appropriated conjugated secondary antibodies Alexa Fluor® 568 goat anti‐rabbit IgG (A11011, Life Technologies) or Alexa Fluor® 488 goat anti‐mouse IgG (A11029, Life Technologies). The immunofluorescence‐mounting medium used was Fluoromont G® (BioNova cientifica). Representative images were taken using a confocal microscope Leica TCS SP5 X (Leica, Barcelona, Spain).

### 2.14 Statistical analysis

Data from qPCR and western blot experiments are expressed as percentage, and presented as mean ± standard error of mean (SEM) relative to control condition (100%); data from immunohistochemistry are expressed as percentage or arbitrary units, and presented as mean ± SEM; data from flow cytometry experiments are expressed as fold increase, and presented as mean ± SEM relative to control condition (1). Data were compared using one‐way analysis of variance (ANOVA) followed by Bonferroni test. All statistical analyses were performed using the GraphPad Prism 6.0 software (GraphPad Software Inc, San Diego, CA, USA) and the IBM SPSS Statistics 24.0 (SPSS Inc, IBM, Armonk, NY) software with two‐sided tests, with a *P*‐value of < .05 considered as statistically significant.

## 3 RESULTS

### 3.1 Hypoxia induces lipid accumulation and CD36 expression in both human and murine hepatocytes

**Figure 1:**
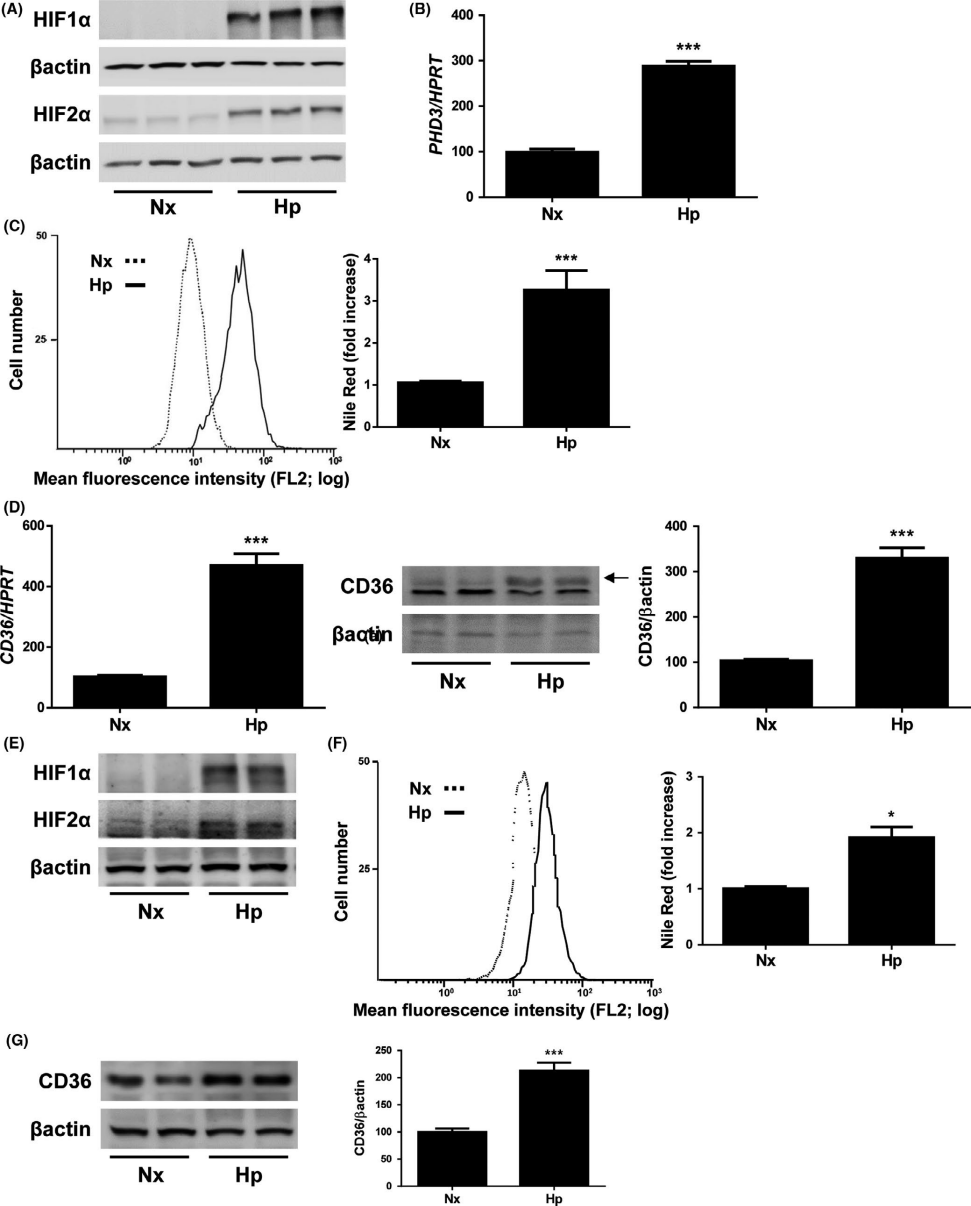
Hypoxia induces lipid accumulation and CD36 expression in hepatocytes. Huh7 cells maintained under normoxic (Nx, 21% O_2_), or hypoxic conditions (Hp, 1% O_2_) in a hypoxia chamber for 36h. A, Representative blots with the indicated antibodies. B, *PHD3* mRNA levels. C, *(left panel)* Representative experiment of Nile Red fluorescence intensity. *(right panel)* Analysis of intracellular lipid content by Nile Red staining. D, *(left panel)* CD36 mRNA. *(right panel)* Representative blots with the indicated antibodies and densitometric analysis from all blots. ***P < .005, Hp vs Nx (n = 4 independent experiments performed by triplicate). AML12 cells maintained under normoxic (Nx, 21% O_2_), or hypoxic conditions (Hp, 1% O_2_) in a hypoxia chamber for 36 hours. E, Representative blots with the indicated antibodies. F, *(left panel)* Representative experiment of Nile Red fluorescence intensity. *(right panel)* Analysis of intracellular lipid content by Nile Red staining. G, Representative blots with the indicated antibodies and densitometric analysis from all blots. *P < .005 and ***P < .005, Hp vs Nx (n = 3 independent experiments performed by duplicate)

To explore the molecular mechanisms involved in the regulation of CD36 by hypoxia, Huh7 human liver cells were maintained under normoxic conditions (Nx, 21%O2) or submitted to hypoxia (Hp, 1%O2) for 36h. To assure that hypoxia was achieved in Huh7 cell cultures, we assessed the expression of HIFα protein subunits by western blot. As Figure 1A shows, HIF1α and HIF2α expression was markedly induced by hypoxia. Accordingly, *PHD3* upregulation, a well‐recognized HIF responsive gene, was found in hypoxia‐exposed Huh7 liver cells (Figure 1B).

Next, we investigated whether Huh7 cells submitted to hypoxic conditions would increase their intracellular lipid content performing Nile Red staining experiments. After flow cytometry analysis, we observed a significant increase in the lipid content of Huh7 cells submitted to hypoxia, compared to those maintained under normoxic conditions (Figure 1C). Interestingly, a parallel increase in CD36 mRNA and protein levels was found in hypoxic Huh7 cells (Figure 1D). Similar findings were observed in mouse hepatocytes (AML12 cell line) submitted to hypoxia in which both intracellular lipid content and CD36 expression were significantly augmented when compared to normoxic cells (Figure 1E‐G).

### 3.2 *Silencing of CD36* attenuates hypoxia‐induced lipid accumulation in liver cells

**Figure 2:**
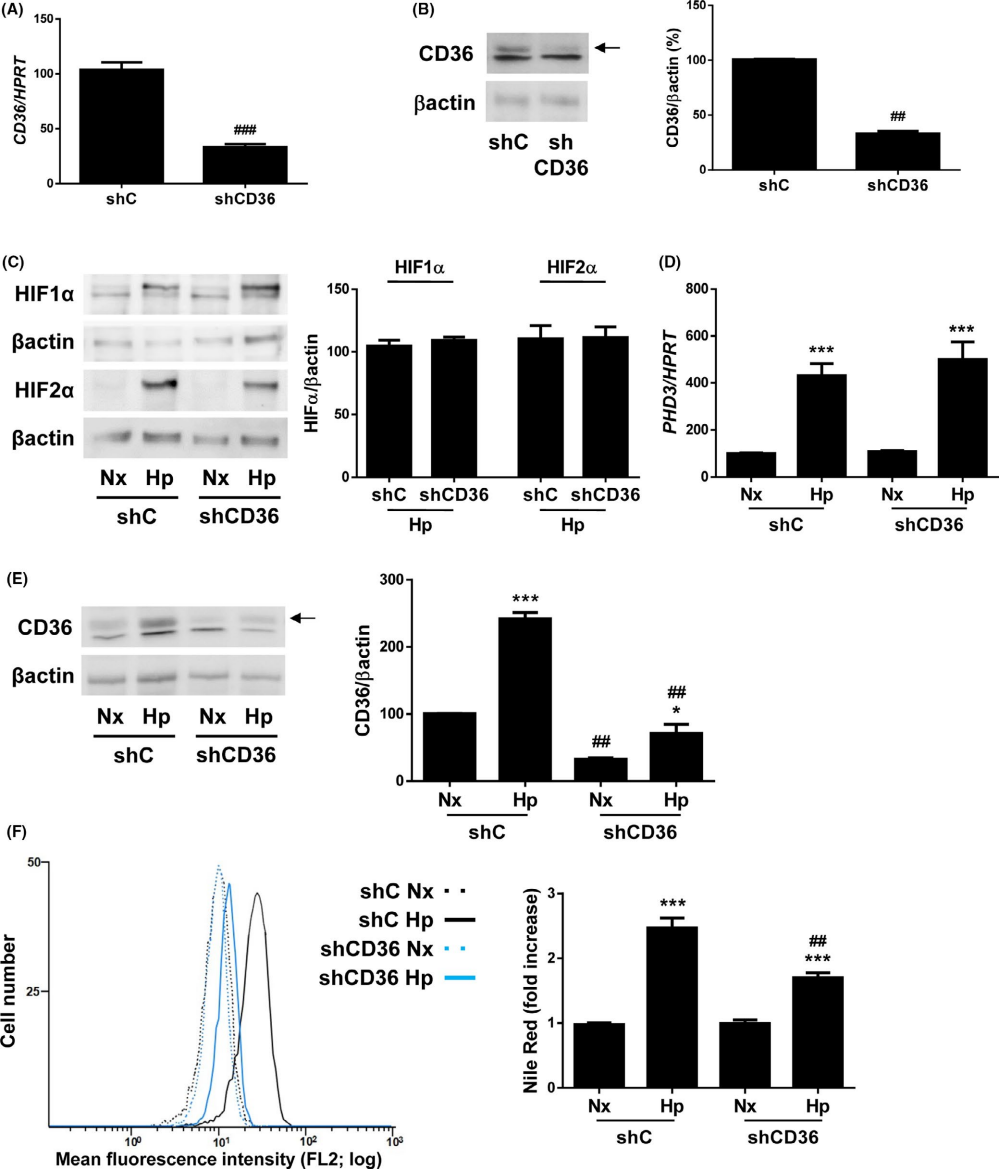
CD36 knockdown attenuates hypoxia‐induced lipid accumulation in human liver cells. Huh7 cells infected with scrambled (shC) or CD36 shRNA (shCD36) lentiviral particles maintained under normoxic (Nx, 21% O2), or hypoxic conditions (Hp, 1% O2) in a hypoxia chamber for 36h. A, CD36 mRNA in normoxia. B, C, and E, Representative blots with the indicated antibodies and densitometric analysis from all blots. D, *PHD3* mRNA levels. F. *(left panel)* Representative experiment of Nile Red fluorescence intensity. *(right panel)* Analysis of intracellular lipid content by Nile Red staining. ^*^ *P* < .005 and ***P < .005, Hp vs Nx; ^##^ *P* < .01 and ^###^ *P* < .005, shCD36 vs shC (n = 4 independent experiments performed by duplicate)

To elucidate whether CD36 is involved in hypoxia‐induced lipid accumulation in hepatic cells, we infected Huh7 cells with scrambled (control, shC) or CD36 shRNA (shCD36) lentiviral particles. With this approach, we obtained an average of 70% decrease of mRNA and protein levels of CD36 (Figure 2A,B). As depicted in Figure 2F, silencing of CD36 significantly reduced lipid accumulation in Huh7 submitted to hypoxia without altering the induction of hypoxia markers (Figure 2C,D) and partly blocking hypoxia‐induced CD36 increase (Figure 2E).

These data propose that CD36 might play a major role in the onset of hepatosteatosis under hypoxic conditions.

### 3.3 *HIF2A* silencing markedly reduces both lipid accumulation and CD36 upregulation in hypoxic human hepatocytes

**Figure 3:**
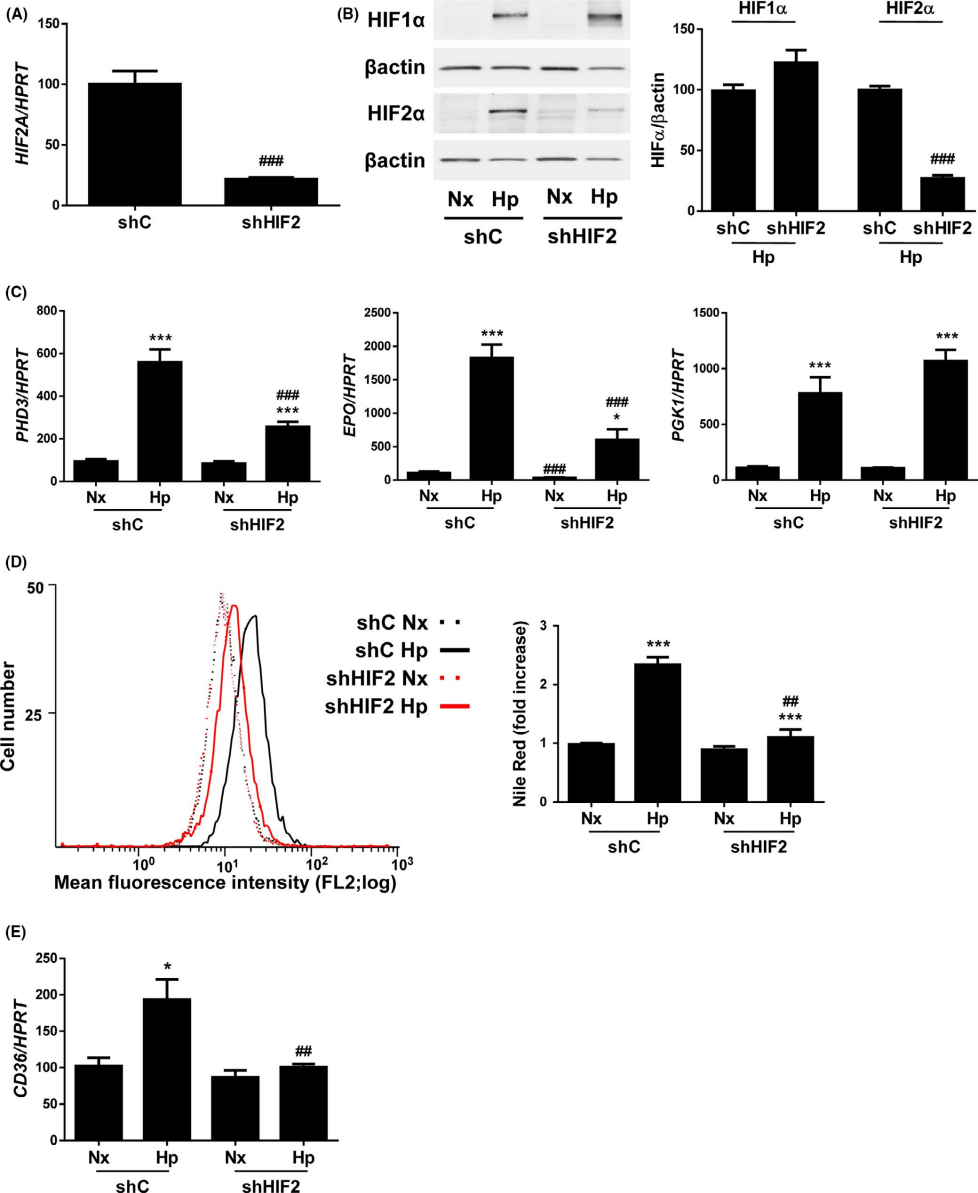
HIF2α silencing reduces both lipid accumulation and induction of CD36 expression in human liver cells submitted to hypoxic conditions. Huh7 cells infected with scrambled (shC) or HIF2α shRNA (shHIF2) lentiviral particles maintained under normoxic (Nx, 21% O2), or hypoxic conditions (Hp, 1% O2) in a hypoxia chamber for 36h. A, *HIF2A* mRNA levels in normoxia. B, Representative blots with the indicated antibodies and densitometric analysis from all blots. C, *PHD3, EPO* and *PGK1* mRNA levels. D, *(left panel)* Representative experiment of Nile Red fluorescence intensity. *(right panel)* Analysis of intracellular lipid content by Nile Red staining. E, *CD36* mRNA levels. *P < .05 and ***P < .005, Hp vs Nx; ^##^ *P* < .01 and ^###^ *P* < .005, shHIF2 vs shC (n = 4 independent experiments performed by duplicate)

We next wanted to determine whether HIF2α might be linked to hypoxia‐induced CD36 upregulation. To this end, we infected Huh7 cells with scrambled (control, shC) or HIF2α shRNA (shHIF2) lentiviral particles achieving a 75% decrease in *HIF2A* mRNA levels (Figure 3A). Under hypoxia conditions, it was observed a reduced HIF2α protein stabilization (Figure 3B), as well as *PHD3* and *EPO* mRNA levels, while the hypoxia‐induced increased expression of PGK1, a major HIF1α target gene, remained unchanged (Figure 3C). Noteworthy, the reduction of HIF2α significantly decreased both lipid accumulation and *CD36* upregulation observed in Huh7 cells submitted to hypoxia for 36 hours (Figure 3D,E). Taken together these data suggest that HIF2α is the responsible for the hypoxia‐induced CD36 upregulation and, ultimately, for the increase of lipid content in hypoxic hepatocytes.

### 3.4 **Lack of HIF2**α** ameliorates NASH features and decreases CD36 content in livers from Vhl^f/f^‐deficient mice**

**Figure 4:**
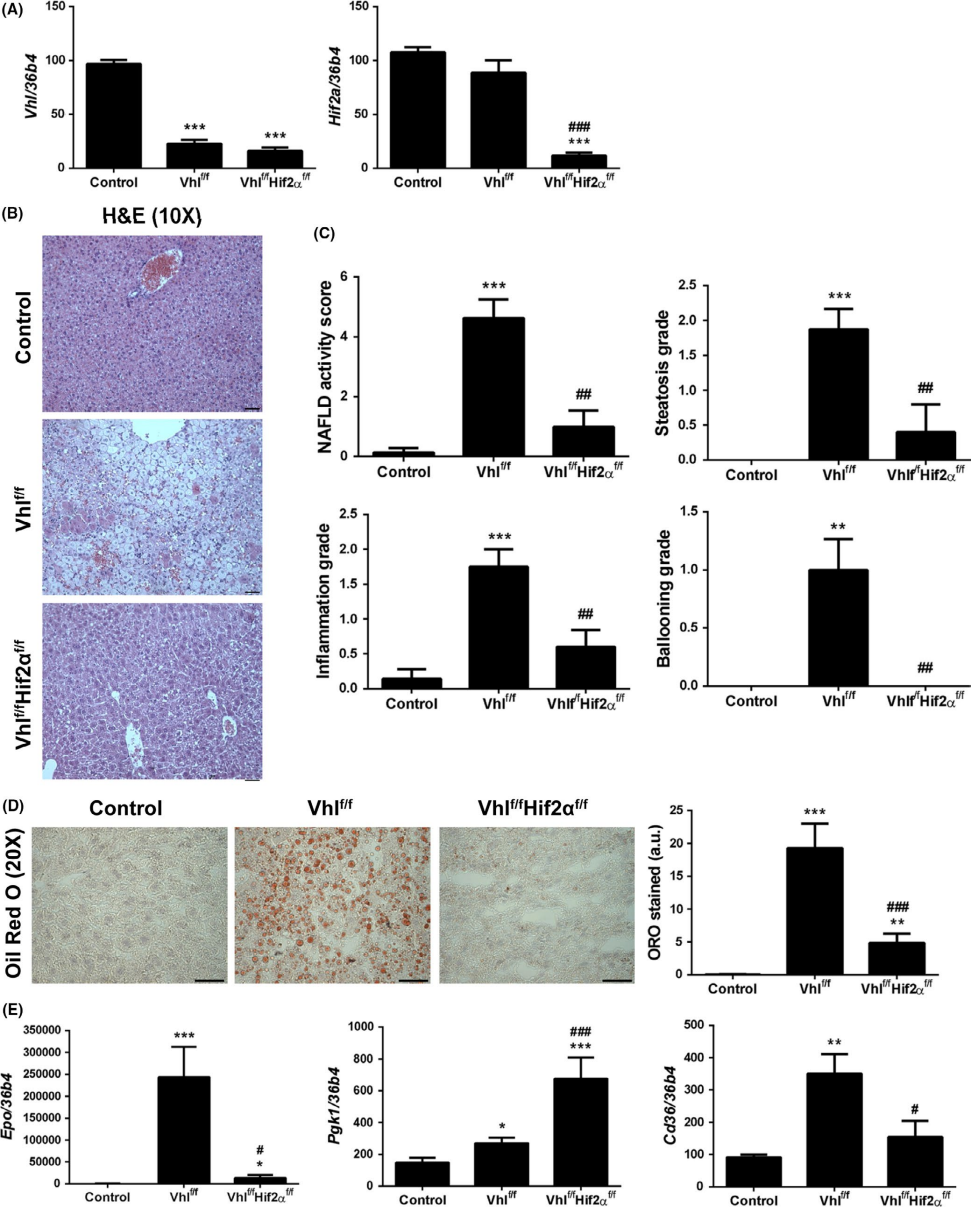
Lack of HIF2α reverts *Vhl* inactivation‐induced NASH. A, Hepatic *Vhl* and *Hif2a* mRNA levels. B, Representative 10X images of haematoxylin/eosin (H&E) staining. Scale bar 100 µm. C, NAFLD activity score, steatosis grade, lobular inflammation and hepatocellular ballooning grade. D, Representative 20X images of Oil Red O (ORO) staining, and its quantification. Scale bar 100 µm. E, Hepatic *Epo*, *Pgk1* and *Cd36* mRNA levels. Experimental groups: Control, Vhl^f/f^‐deficient mice and Vhl^f/f^Hif2α^f/f^‐deficient mice (n = 6‐8 animals/group). *P < .05, **P < .01 and ***P < .005, Vhl^f/f^ or Vhl^f/f^Hif2α^f/f^‐deficient vs Control mice; ^#^ *P* < .05, ^##^ *P* < .01 and ^###^ *P* < .005, Vhl^f/f^Hif2α^f/f^ vs Vhl^f/f^‐deficient mice

To investigate the role of HIF2α ‐CD36 pathways in the hepatic lipid homeostasis in vivo, we used adult Vhl^floxed^‐Ubc‐Cre‐ER^T2^ mice (Vhl^f/f^‐deficient mice) in which *Vhl* gene inactivation leads to an elevated expression of HIFα protein subunits, and Vhl^floxed^Hif2α^floxed^‐UBC‐Cre‐ER^T2^ mice (Vhl^f/f^Hif2α^f/f^‐deficient mice) in which both *Vhl* and *Hif2a* are simultaneously inactivated (Figure 4A).

Histological examination of liver biopsies revealed that control mice displayed a normal histology while Vhl^f/f^‐deficient mice exhibited borderline or definite NASH due to the combined presence of steatosis, inflammation and hepatocyte ballooning. Interestingly, these NASH features were markedly attenuated in livers from Vhl^f/f^Hif2α^f/f^‐deficient mice (Figure 4B,C). Accordingly, an Oil Red O staining revealed that lipid accumulation observed in liver sections from Vhl^f/f^‐deficient mice was higher than in control animals, and was reverted in livers from Vhl^f/f^Hif2α^f/f^‐deficient mice (Figure 4D).

Interestingly, hepatic *Cd36* mRNA levels were significantly higher in Vhl^f/f^‐deficient mice than in control mice, as well as *Epo* and *Pgk1* mRNA levels which are HIF2α and HIF1α‐dependent genes respectively. Noteworthy, in Vhl^f/f^Hif2α^f/f^‐deficient mice, hepatic *Cd36* mRNA levels were significantly lower than in Vhl^f/f^‐deficient mice, in parallel to *Epo* mRNA levels, whereas *Pgk1* gene expression was not repressed but rather it was over‐induced (Figure 4E).

**Figure 5:**
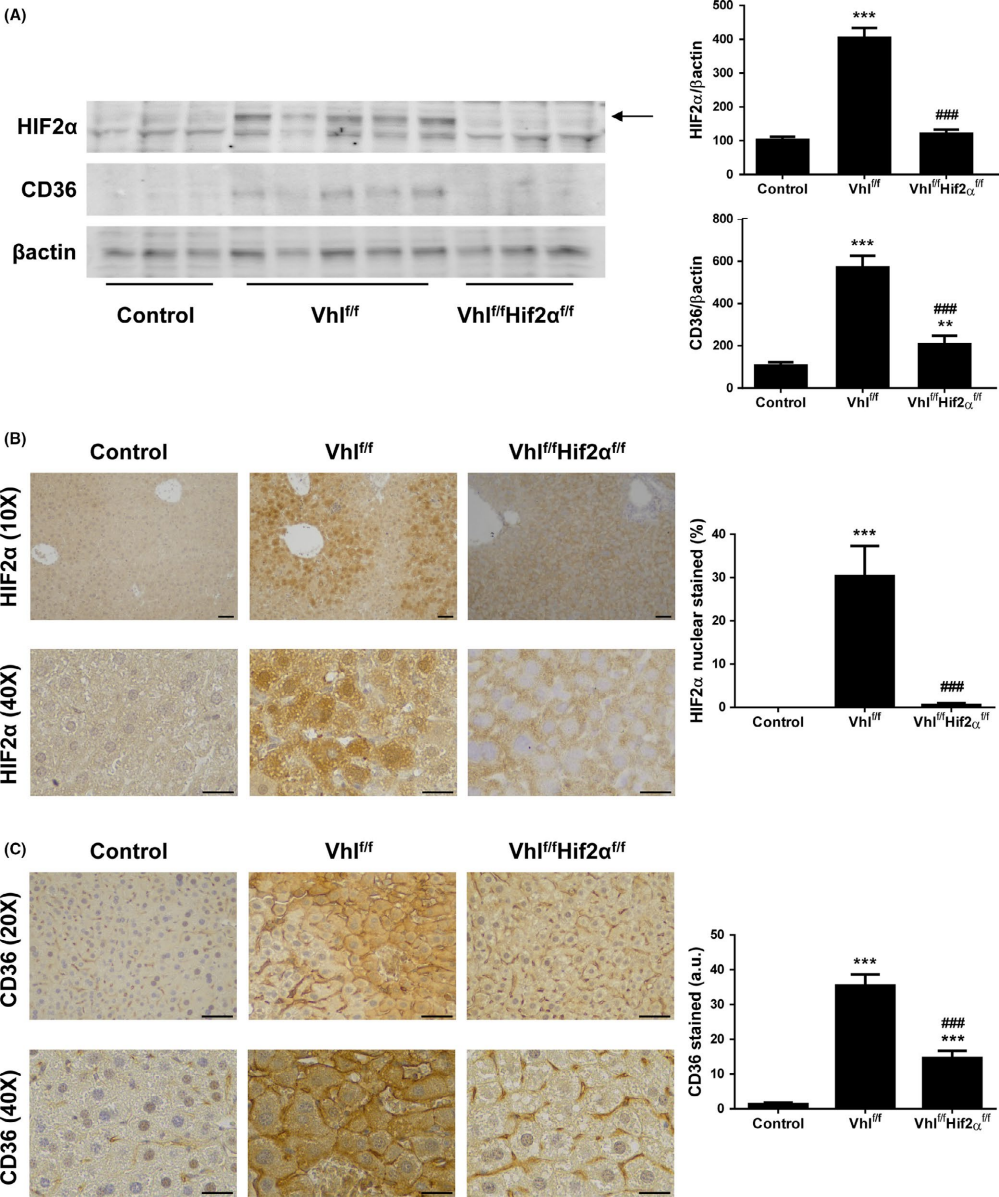
HIF2α deficiency attenuates *Vhl* inactivation‐induced hepatic CD36 overexpression. A, Representative blots with the indicated antibodies and densitometric analysis from all blots. B, Representative 10X and 40X images of HIF2α immunostaining, and quantification of nuclear HIF2α‐expressing cells. Scale bar 100 and 50 µm respectively. C, Representative 20× and 40× images of CD36 immunostaining, and quantification of CD36‐expressing cells. Scale bar 100 and 50 µm respectively. Experimental groups: Control, Vhl^f/f^‐deficient mice and Vhl^f/f^Hif2α^f/f^‐deficient mice (n = 6‐8 animals/group). **P < .01 and ***P < .005, Vhl^f/f^ or Vhl^f/f^Hif2α^f/f^‐deficient vs Control mice; ^###^ *P* < .005, Vhl^f/f^Hif2α^f/f^ vs Vhl^f/f^‐deficient mice

Moreover HIF2α protein expression determined by western blot (Figure 5A) and by immunostaining (Figure 5B) was elevated in the livers of Vhl^f/f^‐deficient mice. As expected, this increase in HIF2α content was not observed in mice with both *Vhl* and *Hif2a* inactivated (Figure 5A,B). Regarding HIF2α immunostaining, nuclear staining was lost in livers from Vhl^f/f^Hif2α^f/f^‐deficient mice with respect to those from Vhl^f/f^‐deficient mice, suggesting that the nuclear signal observed correspond to endogenous HIF2α (Figure 5B). Indeed, we also analysed HIF2α protein content in nuclear extracts by western blot, and we found similar results which indicate that hepatic HIF2α translocation into the nucleus was nearly blocked in Vhl^f/f^Hif2α^f/f^‐deficient mice (Figure S1A).

In parallel, an increase in CD36 protein expression was observed in the livers of Vhl^f/f^‐deficient mice detected by western blot (Figure 5A) and by immunostaining (Figure 5C). In these animals, the intensification of CD36 immunostaining was also enhanced in the plasma membrane of hepatocytes. Indeed, a co‐localization between CD36 and N‐cadherin, a well‐characterized hepatocyte plasma membrane marker,^29^ was observed (Figure S1B). Noteworthy, hepatic CD36 expression was also significantly lower in Vhl^f/f^Hif2α^f/f^‐deficient mice than in Vhl^f/f^‐deficient mice (Figure 5A,C).

Taken together, these findings strongly suggest that HIF2α could play an important role on hepatic lipid homeostasis by regulating the expression and function of the fatty acid receptor CD36 in hepatocytes.

### 3.5 **Expression of HIF2**α** and CD36 is increased within the liver of NAFLD patients**

**Figure 6:**
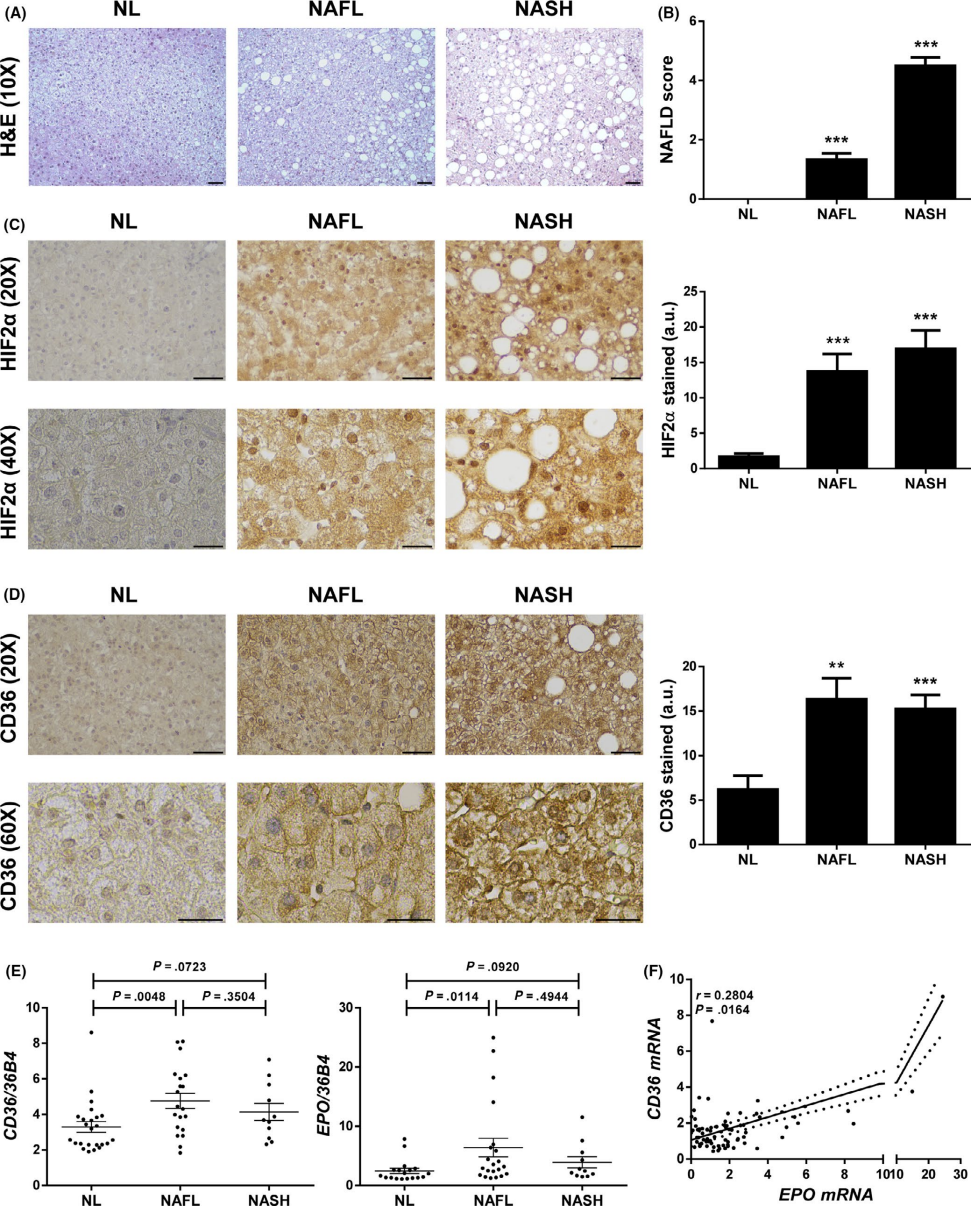
Expression of HIF2α and CD36 is increased within the liver of NAFLD patients. A, Representative 10X images of haematoxylin/eosin (H&E) staining. Scale bar 100 µm. B, NAFLD activity score. C, Representative 20× and 40× images of HIF2α immunostaining, and quantification of nuclear HIF2α‐expressing cells. Scale bar 100 and 50 µm respectively. D, Representative 20× and 60× images of CD36 immunostaining, and quantification of CD36‐expressing cells. Scale bar 100 and 50 µm respectively. E, *CD36* and *EPO* mRNA levels. F, Correlation in the study population of matched mRNA expression levels. Study population: Normal liver (NL) individuals (n = 18), NAFL patients (n = 18) and NASH patients (n = 15). **P < .01 and ***P < .005, NAFL or NASH vs NL

Finally, we wanted to explore whether this link between HIF2α and CD36 exists in human liver as well. Representative haematoxylin/eosin staining liver pictures and the mean of the NAFLD activity score from the study patients are shown in Figure 6A,B. Furthermore, we estimated the hepatic protein content of nuclear HIF2α and total CD36 assessing their expression by immunohistochemistry. A higher expression of HIF2α was observed in NAFLD patients, such in NAFL as in NASH cases, than in NL individuals (Figure 6C). Interestingly, HIF2α immunostaining was also more intense in the nucleus of hepatocytes in NAFLD patients, which is in line with nuclear HIF2 expression upon Vhl inactivation in mouse liver shown in Figure 5B. Moreover in agreement with previous results reported by our group,^23^ CD36 was weakly expressed in liver biopsies from NL subjects, while is markedly expressed at the plasma membrane and cytoplasm of numerous hepatocytes in NAFL and NASH patients (Figure 6D). Further immunofluorescence staining revealed that CD36 co‐localized with N‐cadherin in NAFLD human samples (Figure S1C), which was also found in Vhl^f/f^‐deficient mice (Figure S1B). Finally, we measured hepatic mRNA levels of *CD36* and *EPO,* being the latter one of the best‐characterized HIF2α‐dependent gene targets. Therefore, we used *EPO* mRNA content as a surrogate marker of HIF2α activation. We found that both *CD36* and *EPO* mRNA expression was elevated in NAFLD patients and, interestingly, a significant positive correlation between the mRNA levels of these genes was observed in the entire study population (Figure 6E,F).

## 4 DISCUSSION

This study not only demonstrates for the first time that HIF2α upregulates CD36 expression and function contributing to hepatosteatosis in hepatocytes, but it also provides evidence suggesting that a HIF2α‐induced CD36 upregulation could be operative in vivo and play a relevant role in NAFLD pathophysiology in humans, as we found a significant increase of both HIF2α and CD36 protein content, and an elevated mRNA levels of *CD36* and *EPO*, being the latter a well‐known surrogate marker of HIF2α activation, as well as a marked positive correlation between these genes, in the livers of NAFLD patients. It is important to note that although hepatic mRNA levels of both *CD36* and *EPO* were higher in NAFL than in NASH patients, the differences between these groups were not significant. Likewise, hepatic protein amounts of both CD36 and HIF2α were similar in NAFL and NASH patients indicating that its hepatic expression remains largely stable during histological progression from NAFL to NASH. Taken together, our data strongly suggest that the HIF2α/CD36 pathway could have a pathogenic role in the phase of hepatosteatosis, but further investigation is needed for understanding in depth its impact on NASH progression.

There is a growing evidence indicating that hypoxia contributes to NAFLD development and progression to NASH likely by the effects that HIFs exert on target genes regulating glucose and lipid homeostasis in the adipose tissue, small intestine and liver.^30^, ^31^, ^32^ Regarding the latter, HIF2α‐dependent effects in the liver appear to be linked to its activation level, thus mild HIF2α activation will enhance insulin signalling and fatty acid oxidation whereas potent HIF2α activation will lead to liver dysfunction and steatosis.^33^ In our study, 2 weeks after transgene excision, Vhl^f/f^‐deficient mice showed mild to moderate NASH features with a median NAFLD activity score of 4.5, similar findings to that reported by Qu et al^13^ In particular, these authors observed that livers of mice overexpressing HIFs after 2 weeks of disrupting the *Vhl* gene in hepatocytes displayed NASH features along with a marked increase of *Cd36* mRNA levels. More interestingly, in agreement with our findings showed herein, they also found that the combined inactivation of *Vhl* and *Hif2a* genes in hepatocytes reverted hepatic steatosis and inflammation observed in Vhlf/f‐deficient mice, indicating that HIF2α is a direct regulator of lipid homeostasis in the liver, but whether HIF2α exerts its steatogenic effect by upregulating genes important for FFA uptake in hepatocytes, such as CD36, is still unknown. Shedding light on this issue, our findings provide a robust experimental evidence demonstrating that HIF2α not only upregulates CD36 expression in human liver cells but also its function as FFA transporter because we found a marked decrease of hypoxia‐induced lipid content and *CD36* mRNA levels when we knocked down HIF2α in hepatocytes. Further experimental studies, however, are needed to prove whether the induction of CD36 by HIF2α occurs directly or indirectly, but it is conceivable that HIF2α could activate *CD36* gene expression in human hepatocytes at the transcriptional level as it has been reported at least one putative hypoxia response element consensus site at human *CD36* gene promoter.^24^

To the best of our knowledge, this is the first study demonstrating that hypoxia upregulates CD36 expression and function in hepatocytes, and that *CD36* gene knockdown markedly reduces the increased FFA uptake in these hypoxic hepatocytes. In addition, another novel finding of this study is the concomitant increase in HIF2α and CD36 protein content in the liver of NAFLD patients. In particular, noteworthy, such in NAFLD patients as in Vhl^f/f^‐deficient mice, the increase in HIF2α expression was markedly observed in the nuclei of hepatocytes and that of CD36 was also detected at the plasma membrane of hepatocytes. More interestingly, we observed that histological features of NASH significantly ameliorated in Vhl^f/f^Hif2α^f/f^‐deficient mice along with a marked decrease of both mRNA and protein hepatic CD36 levels, supporting our assumption that HIF2α may contribute to NAFLD onset by upregulating CD36 expression and function in hepatocytes.

The subcellular distribution of CD36 is critical for the regulation of its functional activity as FFA transporter facilitating the uptake and influx of FFA to the cells, remaining functionally inactive at intracellular storage pools and active when translocated to the plasma membrane.^34^ In line with our present findings and reinforcing others we previously reported,^23^ Zhao et al ^35^ have recently confirmed that CD36 is largely located in the plasma membrane of hepatocytes such in NASH patients as in mice with histological features of NASH, providing further evidence indicating the key role of palmitoylation in regulating CD36 translocation to the plasma membrane of hepatocytes. Protein palmitoylation is mediated by a family of palmitoyl acyltransferases (PAT).^36^ In humans, 23 genes encoding PAT have been described so far, whose expression and function could be regulated from transcriptional to post‐translational level.^37^ It is tempting to speculate that HIF2α might induce CD36 expression and its translocation to the plasma membrane of human hepatocytes by upregulating PAT gene expression, thus increasing CD36 palmitoylation which facilitates incorporation of CD36 into plasma membranes, but this hypothesis still remains to be addressed.

In conclusion, this study provides novel evidence indicating that HIF2α upregulates CD36 expression and function in hepatocytes, and could contribute to the onset of hepatosteatosis. Unveiling the molecular mechanisms underlying the HIF2α‐dependent CD36 upregulation in human hepatocytes may have notable implications for the development of new pharmacological therapies inhibiting the HIF2α/CD36 pathway trying to attenuate the excessive fat accumulation within the liver and its detrimental effects on the outcome of NAFLD.

## 5 CONFLICT OF INTEREST STATEMENT

Authors have not conflict of interest.

## Supporting information

Fig S1

Supplementary Material
