# Supplementary figures and images for: Hypoxia‐inducible factor 2α drives hepatosteatosis through the fatty acid translocase CD36
Source: Liver Int. 2020 Jun 10;40(10):2553–67. doi: 10.1111/liv.14519 (PMC7539965; doi:10.1111/liv.14519)

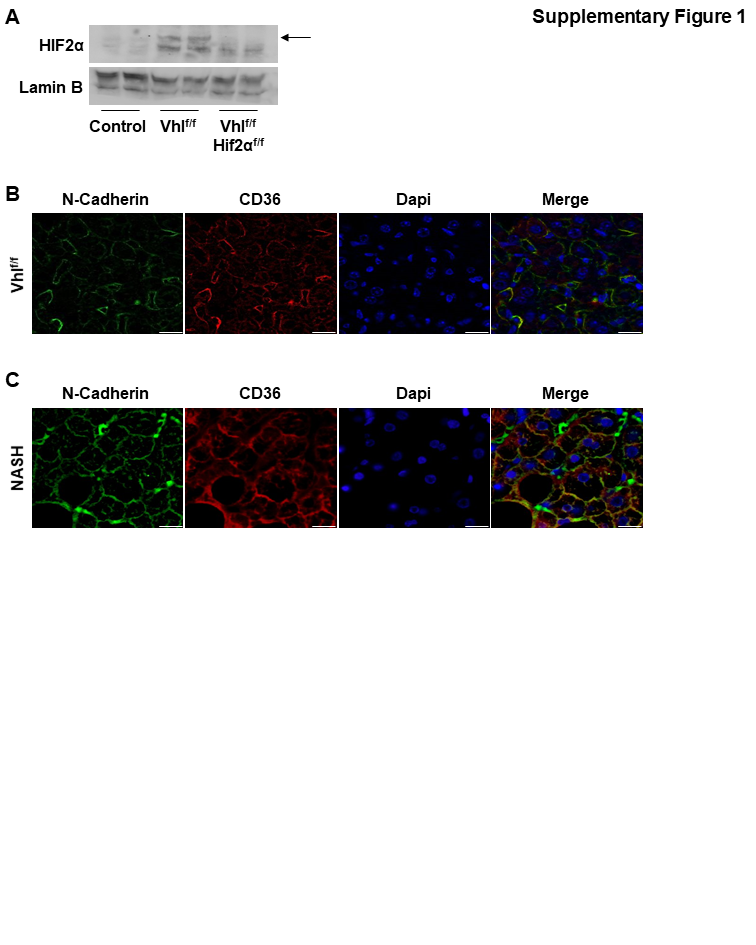

Supplement: Supplementary file 1 — Fig S1 [file LIV-40-2553-s001.tif]
