# Supplementary material for: Hypoxia‐inducible factor 2α drives hepatosteatosis through the fatty acid translocase CD36
Source: Liver Int. 2020 Jun 10;40(10):2553–67. doi: 10.1111/liv.14519 (PMC7539965; doi:10.1111/liv.14519)
Supplement: Supplementary file 2 — Supplementary Material [file LIV-40-2553-s002.docx]

**Supplementary Figure 1. A**. Representative blots with the indicated antibodies of liver nuclear extracts. Experimental groups: Control, Vhl^f/f^ deficient mice and Vhl^f/f^Hif2α^f/f^ deficient mice (n=4-6 animals/group). **B**. Representative 40X N-cadherin and CD36 immunofluorescence in livers from Vhl^f/f^ deficient mice. Scale bar 50 µm. **C**. Representative 40X N-cadherin and CD36 immunofluorescence in livers from NAFLD patients. Scale bar 50 µm.
